# Virtual Expeditions Facilitated by Open Source Solutions Broaden Student Participation in Natural History Research

**DOI:** 10.1093/icb/icac065

**Published:** 2022-06-06

**Authors:** Richelle L Tanner, Talia Y Moore

**Affiliations:** Environmental Science and Policy Program, Chapman University, Orange, CA 92866, USA; Mechanical Engineering, Robotics Institute, Ecology and Evolutionary Biology, Museum of Zoology, University of Michigan, 2505 Hayward St, Ann Arbor, MI 48109, USA

## Abstract

From its genesis in the Victorian era as an activity for the elite to today's emphasis on “Big Data” and continuous monitoring, natural history has a prominent role in scientific discoveries for many fields. However, participation in field expeditions is limited by funding, space, accessibility, and safety constraints. Others have detailed the active exclusion of minoritized groups from field expeditions and harm/discrimination faced by the few who do participate, but we provide one solution to broaden opportunities for participation in natural history: Virtual Expeditions. Virtual Expeditions are broadly defined as open source, web-facilitated research activities designed to analyze bulk-collected digital data from field expeditions that require visual human interpretation. We show two examples here of their use: an independent research-based analysis of snake behavior and a course-based identification of invertebrate species. We present a guide to their appropriate design, facilitation, and evaluation to result in research grade data. We highlight the importance of open source technology to allow for longevity in methodology and appropriate quality control measures necessary for projects that include dozens of researchers over multiple years. In this perspective, we specifically emphasize the prominent role that open source technology plays in making these experiences feasible and scalable. Even without explicit design as broadening participation endeavors, Virtual Expeditions allow for more inclusive participation of early career researchers with specific participatory limitations. Not only are Virtual Expeditions integral to the large-scale analysis necessary for field expeditions that generate impossibly enormous datasets, but they can also be effective facilitators of inclusivity in natural history research.

## Bringing natural history research into the modern age

Historically, observational records of plants and animals provided the fodder for developing fundamental theories in evolution and ecology (Kingsland 2005; Sunderland et al. 2012; Raby 2017). More recently, these same organismal data have been revealing complex and emergent patterns at the interfaces between ecology and evolution (Schmidly 2005). Despite their clear and lasting importance, natural history data have always been challenging to collect and organize (Stork et al. 2019).

Victorian-era naturalists on biological field expeditions collected as much data as possible in a short amount of time through drawings, specimen collections, and written descriptions (Camerini 1997; Levine 2003). Because there was so much information to document during the voyage, interpretation largely began after the voyage amongst groups of colleagues. With the invention of new sensors (e.g., radio telemetry, digital cameras, GPS, accelerometers, and microphones), the volume of data collected during field expeditions is increasing exponentially. Because of the ever-increasing and overwhelming volume of data, many of the findings from recent expeditions are still waiting to be interpreted. In other fields, “Big Data” approaches leverage automated tools to organize and interpret many of these datasets (Davis and Grayson 2007). However, automation, especially machine learning, relies on having informative baseline expectations to perform pattern matching (e.g., Valletta et al. 2017). Thus, these tools are largely incapable of making novel discoveries or working with highly variable data. Additionally, these tools can be particularly difficult to use with photographs and videos because they may be easily fooled by disruptions in learned patterns (Hendrycks et al. 2021). Visual human observation remains the gold standard for spotting partially occluded individuals, identifying species, and describing behaviors (Gaston and O'Neill 2004).

To address the growing gap between the rate of data collection on field expeditions and the ability of automated tools to analyze these data, particularly photographic data, we developed Virtual Expedition undergraduate research experiences (UREs). While there are many existing methods for post-field expedition data processing, we have identified one set of tools currently under-used by biologists: open source solutions. The purpose of this perspective is to highlight open source tools for use in this application, which grew from a discussion fostered by the SICB 2022 “Open Source Solutions in Experimental Design” Symposium, and to encourage colleagues to explore the adoption of open source methods in their own research and classroom environments. We demonstrate the implementation of the Virtual Expedition framework and the role of open source tools in their administration through two different case studies: a course-based undergraduate research experience (CURE) to sort and identify brackish marsh invertebrates from microscope photographs and an apprenticeship-based research experience to digitize snake anti-predator behaviors from video data. These case studies were developed independently from one another yet converged on the majority of their open source implementation traits in their administration. Therefore, we felt that describing the Virtual Expedition format would be useful to those hoping to broaden participation in this area of STEM education.

First, we trained experts how to collect useful data during their in-person expeditions or relied on existing government protocols (i.e., developed by agency labs, such as the US Geological Survey or the National Oceanic and Atmospheric Administration, for internal quality control) to generate uniform, well-annotated data. After the in-person expedition, we recruited and trained undergraduate students to analyze the data, access primary literature, form and test hypotheses using the data they generated, and present their findings. Student-generated data had high accuracy (i.e., passed quality assurance and quality control thresholds at the same rate as trained professionals), likely due to careful examination with fewer time constraints and access to keys and ethograms. In contrast, interpretation during in-person expeditions often relies on the individual's expertise and rapid interpretation (McBride et al. 2012). The Virtual Expeditions have resulted in several important natural history findings, including peer-reviewed literature with student authors and contributions to local and state agency management plans (Davis Rabosky and Moore et al. 2021). Each case study here has supported the analyses of thousands of photo/video observations, representing a portion, but not all, of the samples contained within the associated field expedition. On average, over 1000 student hours were logged for the invertebrate identification Virtual Expedition per academic term. Students who participated in Virtual Expeditions were more demographically diverse than the members of the in-person expeditions who collected the data. Virtual Expeditions have proven to be a useful way to accurately analyze natural history data using open source tools while serving as a gateway into more research opportunities for participants.

## Virtual Expeditions have scalable formats

There are two models of undergraduate research: the CURE and the apprenticeship-based URE (Kardash 2000; Auchincloss et al. 2014). While they share characteristics, the main difference is the instructor–student ratio, which influences the relationship between mentor and mentees (Laursen et al. 2012). This distinguishing characteristic also controls scalability, making it important to identify the goals of a Virtual Expedition before selecting a format (see Lei and Chuang 2009 for a cost-benefit analysis of research experience types). Traditionally, the URE model facilitates greater student independence, particularly in hypothesis development and analyzing data to address student research questions (Hunter et al. 2007; Linn et al. 2015). However, there are ways to mitigate the larger scale of a CURE to facilitate similar learning objectives. If the instructor aims to impact a greater proportion of the undergraduate student population, a CURE model is advantageous (Auchincloss et al. 2014; Kinner and Lord 2018). The higher mentor–mentee ratio can be overcome with strategic use of peer mentoring using a dyad structure, which has been shown to maintain low-stress accountability (Fantuzzo et al. 1989). If the instructor is able to recruit more mentors to aid in a Virtual Expedition (i.e., postdoctoral fellows or graduate students), an URE model may become feasible at a larger scale and foster deeper connections between students and facilitators (see multi-mentor models in Lopatto et al. 2014).

Whichever model is selected, we advocate for incorporating four main components into the Virtual Expedition research experience. In our own case studies, each of these components is feasible regardless of course size, but implementation within a CURE requires additional scheduling and data management.

1. *Students must receive uniform, comprehensive training for the research task*, including samples that represent the diversity of potential analyses they will be required to perform (see onboarding documents in Supplementary Materials). Students must also receive the opportunity during this training to work through sample data independently with feedback, not just in a follow-along tutorial (i.e., “learning by doing,” see Reese 2011). It is important that this training includes a description of the data set generation methods and sources, emphasizing the significance of open source methods and sharing. For example, the snake behavior URE requires students to watch videos of diverse behaviors they will be expected to score, work through their answers with each other, and then confirm with the mentor to increase confidence and accuracy in IDs. In the invertebrate identification CURE, students are required to create dichotomous keys to distinguish between the top 10 most commonly encountered species in the sample set and explain their process for finding identifying traits from the literature. This component is ideally available in a recorded and written format to allow students to reference back to it. This is especially important for maintaining the availability of information in a virtual format (Cohen et al. 2021).
2. *Students must have the opportunity to develop their own hypotheses from the dataset*, even if there is a broader goal identified by the field research team. However, this must be a guided process so that students may build the requisite confidence to make their own discoveries (Lee 2012). There are diverse methods of guiding scientific discovery, but we advocate for weekly mentor–mentee meetings (URE), or peer mentoring paired with at least biweekly short instructor meetings (CURE) in the early stages of hypothesis development. Students can build confidence in hypothesis generation by discussing observations from their own datasets and asking questions about published research throughout the semester with their mentors. This component is ideally linked with a final presentation to researchers outside of the immediate project.
3. *Students must participate in project meetings with structured elements for professional development*. We advocate for students to experience a diversity of typical “lab meeting” styles, including facilitated journal clubs, informal “meet and greets” with individuals in diverse career paths (i.e., career panel or visiting researcher talk), group statistics lessons and troubleshooting with code, and science communication workshops. These types of interactions provide exposure to many skills and career paths beyond their undergraduate courses and the multiple facets of being a scientist beyond research (Jelks and Crain 2020). The snake behavior URE was conducted in the context of the University of Michigan Undergraduate Research Opportunity Program, which provided seminar-based workshops to aid in professional development. It can also be helpful to create and review mentor–mentee contracts for the work, which allows students to communicate their needs and expectations for the project and for mentoring (e.g., Pfund et al. 2005). This is also an opportunity for students to realize that research experiences include significant professional development and “soft” skills, such as interpersonal communication and organization, which are often undervalued yet simultaneously disproportionately held by individuals who fit within existing societal norms (e.g., native English language, meritocracy-based communication, and neurotypical experiences). For a discussion on the value of interpersonal skills in undergraduate education, see Hora et al. (2018). We advocate for completion or contract grading (see Hiller and Hietapelto 2001) if this is being presented as a CURE or an URE for a letter grade, because we strongly believe research experiences should have confidence building as their primary goal.
4. *Students must prepare a final presentation*, preferably in the format of an academic conference presentation. In the invertebrate CURE, students frequently listed a primary professional goal as “seeing a research project from start to finish.” This is supported by the efficacy of project-based learning in the classroom setting (Garnjost and Lawter 2019). Being responsible for evaluating their hypotheses provides another opportunity for independence and increases satisfaction with the research task. We also advocate for including role models outside of the institution or lab where the research is being conducted, particularly focusing on collaborators directly involved with the instructor. This reveals how important networks of collaborators are for job prospects and career-long relationships, and gives students the sense that they are entering into a rich network that welcomes their ideas (Hernandez et al. 2018).

## Communication and built-in redundancy are key to data collection and management

In this section, we refer frequently to “field researchers” and “student researchers” to distinguish between those involved in field data collection and virtual data analyses, respectively.

### In-person data collection

In-person field expeditions remain one of the most efficient strategies to collect natural history data on a variety of species for a specific location. We recommend contacting natural history museums (e.g., the snake behavior URE was in collaboration with the University of Michigan Museum of Zoology Division of Herpetology Expeditions) and governmental agencies (e.g., the invertebrate identification CURE was in collaboration with the US Geological Survey Invertebrate Zoology Laboratory) to coordinate with planned or ongoing field expeditions. To maximize the mutual benefit to researchers at all stages of data collection and analysis, it is important to determine the data collection logistics of the expedition and to explain the broader context and significance of the intended study. Understanding the day-to-day routines of the field expedition can help design methods to minimize the added hassle of collecting additional data types and learn how expedition leaders plan to organize their data and keep track of their records during the expedition. This information is essential for designing compatible data collection methods so that downstream virtual data analyses can run smoothly.

In large, museum-associated excursions, each field researcher may be planning to collect data for their own projects as well as for many other projects in which they do not plan to participate in the future. There is usually a series of formal or informal training sessions for various types of data collection. We recommend creating a training program for all field researchers, including an interactive demonstration, a video tutorial, and a written document that is available both on the cloud and as a laminated printout. If the field researchers understand how the data will be analyzed by student researchers and the main challenges of the analyses, the data will be of higher quality.

It is essential to have a comprehensive data management plan. Ensure that everyone is on the same page about how to record, label, save, and backup the data. If possible, automate this process before the expedition. For example, ensure that all cameras are synchronized to a specific time zone and date. This can inform the recovery of data organization if something goes wrong in the future. If paper notes are taken, take photographs with a camera or phone and save them to multiple devices. If connection to the internet is available, use automated software to upload data to Cloud-based storage solutions (e.g., Google Drive, Dropbox, Box Sync) or develop an app interface to collect data (e.g., Rife and Poland 2014). If connection to the Internet is unavailable, provide multiple solid-state hard drives that can save redundant copies of the data and pack them in separate pieces of luggage. Immediately upon return from the field, examine the data, back up to a Cloud-based storage solution, and check for any inconsistencies. If there are any questions from student researchers, it is best to ask the researchers that undertook the field expedition while the trip is still fresh in their minds.

## Virtual data analysis administration

Requiring student researchers to participate in data management has several benefits. First, it helps them understand how data management facilitates efficient and reliable access to data for downstream analyses. When both field and student researchers are familiar with the data management system, it is easy to ask for a second opinion on a specific item, even when everyone is working remotely. Second, it can help student researchers track their own progress, report progress to supervisors, and minimize redundant observations. When student researchers report progress during weekly meetings, it can help develop a sense of friendly competition that motivates student researchers to keep up with the pace of their peers. Third, student researchers can be encouraged to share data to test hypotheses, thereby participating in collaborative research. This act of data sharing helps student researchers understand the significance of their contribution to the overall project goals.

Storing and organizing data in the cloud allows student researchers to access the data from any physical location. Additionally, it allows for asynchronous participation in the research experience, further broadening who can engage with the data (e.g., other time zones, home responsibilities, or personal preference in working hours). However, providing editing access to many individuals will inevitably result in accidental deletions. In the case studies presented here, we found a few key strategies to mitigate data disasters. For more discussion of data management practices in the classroom, and with respect to accessibility in those spaces, see Mooney et al. (2014) and Reisner et al. (2014).

1. *Create a read-only repository of raw data that the student researchers cannot access*. Then, make a copy of the repository that the student researchers can edit. After each semester, copy the data generated by the student researchers into the read-only repository.
2. *Provide a personal subfolder for each student or student pair*. This will allow students to temporarily store data that they are in the process of analyzing. Generally, when personal subfolders are used, accidental deletions are limited to each student's own folder and do not affect others.
3. *Use automated software that uploads data generated on a laptop to the cloud*. For example, Dropbox has a desktop sync application that can selectively synchronize the student's subfolder to their desktop. This ensures that a file with any amount of progress that is saved in the folder will be uploaded to the cloud. If a student researcher's computer breaks down during the semester, having the data automatically upload to the cloud can help preserve the data and allow the student to maintain progress. This also helps to mitigate the loss of data when researchers depart quickly at the end of the semester.
4. *For larger groups of student researchers, require data reporting in parallel formats*: once in their own spreadsheet in their subfolder, and once in a form that automatically uploads time-stamped results to a read-only spreadsheet.

If the data are associated with individuals that become vouchered museum specimens, natural history museums may be able to assist with the storage and management of these large datasets. As museums are digitizing more of their collections (Heerlien et al. 2015), they are starting to create the infrastructure to link diverse forms of digital data to individual specimens. There are also many open source platforms for data management that the reader may wish to explore, which are far more complex than described here (e.g., Crosas^2011^). Describing to student researchers how linking multiple forms of data by specimen will facilitate future museum-based integrative research can help them appreciate the long-lasting significance of their research efforts and the benefits of collaborative data-sharing.

## Open source software integrates quality control

A major benefit of the Virtual Expedition program is the relatively few hardware and software requirements. The only piece of hardware required is a personal computer that should be available to each student. Because of this hardware-independent model, these projects have successfully endured, and even thrived, during the COVID-19 pandemic. Software requirements will depend on the type of data collected. Pre-processing the dataset could potentially be useful, but because of the large datasets involved, this should only be undertaken if the tools are completely automated and there is a significant benefit to the student researchers. For example, automated color correction for visual data (i.e., photographs or video) may be helpful to implement prior to analysis.

Software must be compatible with Mac, Linux, and PC systems to allow for participation by any student. Consider extending this requirement to machines like Chromebooks, which cannot run the open source software, *R (www.r-project.org)*, but are one of the cheapest computing machines available to students. Open source software is highly preferred because of the documentation and continuous updates needed to maintain functionality throughout operating system upgrades. Even if paid software is available through the university, subscriptions are not guaranteed over time. Also, ensure that the software continues to be supported by the developers, as bugs and backwards compatibility issues can be detrimental to progress. Open source software supported by a rich online community ensures transparency in methodology and access to troubleshooting help (Von Krogh and Von Hippel 2006, also see community standards of the Open Source Initiative at www.opensource.org).

Open source software also aids in quality control measures, which are extremely important for studies that involve many students with no training outside of the project. Some open source software can be customized to accommodate parallel analyses or automated field naming. Clear documentation and the ability to manipulate source code to fit specific projects is a huge asset in designing easily interfaced virtual workspaces (e.g., Blumstein et al. 2006; Pastell 2016; Chang 2018). Virtual Expeditions are designed for high turnover rates, which facilitate increased reach but can also introduce error into the research data due to lack of continuity and overlap between student groups. To increase the research-grade viability of data generated by Virtual Expeditions, we recommend a series of quality control and quality assurance measures.

Ensure that there is a standardized methodology established for data analysis to create compatible results, including quality control measures (Tyson 2019). In the materials provided to students, objective and discrete categories for identification can reduce error (e.g., presented in a drop-down menu format or multiple choice). If starting this program anew, a pilot program with a smaller group can be helpful, including both novices and experts, to develop the methodology (see, e.g., Davis Rabosky et al. 2021). It is important that the categorization method rely on objective features as much as possible. For example, with the snake behavior URE, descriptions of shape, orientation, and motion were preferred over the more traditional subjective descriptions of defensive, offensive, and exploring. By relying only on objective features, rather than subjective interpretations, novices can provide accurate data with high reliability.

Designing for objective and discrete features can also reduce data processing (e.g., if characters are capitalized differently or words are spelled incorrectly). This can be achieved by using drop down menus in software or keys used for data collection. For example, students in the snake behavior URE must select from a pre-populated list of options. In the invertebrate identification CURE, a list of taxonomic classes as column headers on a printed datasheet is provided for initial binning during data collection. Wrapping data collection in a graphical user interface (can be as simple as a Google Form) allows the instructor to control the file naming format, ensuring uniformity. Collecting data as responses rather than direct entry gives the opportunity to collect researcher identity and time stamps for additional transparency. In a script-based collection method, file names can be automatically generated using a few simple lines of code, instead of relying on students to correctly name files.

With repeated measures, potential bias can be detected by comparing the mean observation for each species/individual across students and the mean observation of each student across species/individual. An extra step of quality assurance that was performed in the invertebrate identification CURE is a 10% assessment by a trained lab technician of all samples for each student. In this process, the lab technician assessed whether the student completely captured all of the invertebrates within a subsample, whether the subsample was representative of the entire sample, and the accuracy of the final invertebrate identifications. If error rates exceeded a threshold, the data generated by that student was reprocessed by another individual. This extra step was incorporated into the invertebrate identification CURE because we were working with a government lab with the quality assurance and control protocol already in place. We feel this extra step is worthwhile for researchers using larger datasets that cannot be individually checked by a trained researcher because it significantly cuts down on oversight time while ensuring high accuracy and data quality (Stark et al. 2001; Cox et al. 2012).

The last component of high-quality assurance is instilling confidence in students to be experts in the research project and seek collegial help outside of the immediate research team. We must continuously remind students that they are becoming knowledgeable and their participation in a collaborative process will lead to more reliable and consistent data (Tynjälä 1999). Additionally, there is evidence that individuals who are learning tend to generate more accurate data than individuals with ample experience in ecological identification because they do not rely on assumptions and biases (McBride et al. 2012). Instead, they more frequently seek out the advice of others and additional resources. In order to facilitate this learning process, we need to lead by example in asking colleagues for help when we are not sure about an analysis and provide the resources for students to contact our colleagues for help directly. We find that messaging platforms like Slack and Discord break down social barriers perceived by students to more readily facilitate informal interactions of this nature. We also advocate for empowering students to reach out beyond the instructor's network to other academic institutions like museums and universities, but also community science programs and traditional ecological knowledge holders. Even having a conversation with students about personal anecdotes in which the instructor sent an unsolicited email to a potential collaborator can assist in empowering students to make those same connections.

## Challenges for long-term implementation

There are four logistical and philosophical challenges we see in running a long-term Virtual Expedition. First, software and even hardware updates over the years will disrupt existing data collection pipelines and require adjustments in training materials. However, this is a challenge for all types of research and can be mitigated by annual minor updates to avoid complete upheaval of protocols every five to ten years. It may also be advantageous to build in training materials that introduce students to this inevitable challenge of science and create a learning experience within it. Open source solutions head off this problem with copious documentation and online communities of practice (e.g., projects within the National Ecological Observatory Network supported by the National Science Foundation; https://www.neonscience.org).

Second, because students come from a diversity of backgrounds and skill sets, it is necessary to teach in a way that allows them all to arrive at the same training level. This is, again, a common challenge in STEM pedagogy and is solved by establishing basic common ground, even if students indicate they are more advanced. One aspect of this challenge is unique to Virtual Expeditions because the use of open source software (and sometimes hardware) is required, and computer programming can be perceived as daunting for some students. We have found the “Data Analysis and Visualization in R for Ecologists” free, open source course offered by Data Carpentry to be an excellent and approachable primer for students to enter this world on a level playing field (https://datacarpentry.org/R-ecology-lesson/).

Third, students (and instructors) may perceive the dataset to be insurmountable and lose hope that meaningful results will be achieved at the end of a research experience, especially if the Virtual Expedition has run for multiple years on the same project. We believe our structured, four-component format can help alleviate this stress: by having students form their own hypotheses that can be addressed with the available data (self-generated, or from collaborators and past participants) and then presenting it to a formal audience, they gain a sense of accomplishment even if the broader project continues past their participation. [For analogs of this experience in classroom settings, see project- and inquiry-based learning endeavors in Lee (2012) and Garnjost and Lawter (2019).] Optionally, this can also be an opportunity to recruit students to stay on for additional research experiences or to be involved in the writing process for the results of the Virtual Expedition, providing yet another avenue of professional development and a “peek behind the curtain” into the reality of academic publishing. Having continued participation of alumni in the research project can be an opportunity for community building, not a barrier to enthusiastic participation.

Lastly, it is imperative that Virtual Expeditions have a long-term project coordinator who is responsible for the big picture management of data, progress, and seeing it through publication (Lopatto et al. 2014). This increases the instructor's necessary investment into the Virtual Expedition (time, money if hiring staff), but the data from Virtual Expeditions are intended to be usable, high-quality research data and should be treated as such in the allocation of resources in a lab.

## Virtual Expeditions broaden participation in science

Victorian naturalists are needed to be culturally and financially well-off to devote countless hours and large sums of money to their biological pursuits (Adler 2011). When data collection is only accessible to a small, relatively homogeneous group of people, the findings are likely to be filtered through a narrow perspective, often omitting local, indigenous knowledge (Zuroski 2017; Lee 2020; Tanner et al. 2021). Currently, field expeditions remain inaccessible to many groups of people, for many of the same reasons (Merenlender et al. 2016; Rushworth et al. 2021). Fieldwork continues to be dangerous (especially for those who are not white, cis-gender, heterosexual men), expensive, and requires a substantial time commitment (Nelson et al. 2017; Demery and Pipkin 2021; Lawrence and Dowey 2022). With the increased cost of college tuition and the pressure to graduate on time, few undergraduate students have the opportunity to experience the natural world that they learn about in class. Virtual Expeditions make natural history research accessible to students who have physical disabilities that prevent them from attending expeditions, who are immigrants (legal or undocumented), who must work part-time, or who must help care for family (for more examples of virtual fieldwork, see Stokes et al. 2012, Getchell et al. 2010). Perhaps most impactful is the opportunity for Virtual Expeditions to be run almost completely asynchronously, allowing participants to work at their own pace and still receive community support from peers. Collaborating with campus-based undergraduate research opportunity programs may also provide funding to hire students in lieu of course credit, making Virtual Expeditions a part-time job.

Natural history is an enticing topic for many students starting out in biology because it is often experienced outside of the classroom. UREs that focus on telling stories about nature, even if they are rooted in technical practices, have the potential to be successful in broadening participation in science (for examples in the classroom, see Tobler et al. 2021; Valle et al. 2021). Additionally, using open source methods and practices further democratizes the science and demonstrates to students that the instructors have a vested interest in broadening participation more generally.

To foster a welcoming and inclusive environment, it is essential to consider how the history of white masculine dominance in academia has resulted in cultural norms, stereotypes, and expectations that lead members of underrepresented groups to feel alienated from participation in research. The collaborative and open source nature of Virtual Expeditions works to break down perceptions of scientists, such as individualism, competition, and meritocracy, that have been shown to discourage women and underrepresented groups from pursuing STEM careers (Tran et al. 2011; Rainey et al. 2018; Wegemer and Eccles 2019). Participation in collaborative research experiences also builds cognitive, social, and professional networks that support the retention of underrepresented groups in STEM (Hunter et al. 2007; Vieyra et al. 2011; Hilts et al. 2018). Building research networks has the additional benefit of helping students appreciate the social value of STEM research and learn about diverse career opportunities available in the field (Bonous-Hammarth 2000; Brown et al. 2018).

The CURE has been offered for three terms (Fall 2020, Winter 2021, and Spring 2022) at two institutions during the COVID-19 pandemic. Of the 48 undergraduate student participants, eight participated from a different university than where the CURE was hosted. After the program, >54% were known to continue research in other labs or the lab of the PI. For example, one student was recruited directly into a related field before graduation and deferred their acceptance of the job to be a supervisor for the CURE for another term to gain further independent research skills (student assistant coordination, data analysis, biochemistry assays, and manuscript preparation) that would eventually help them apply to graduate school. One student was hired to a project within the same department as the CURE after graduation, deferring graduate school in a different discipline in order to first pursue their interests in fundamental biology research, which were uncovered in this CURE (this was their first research experience).

The URE has been offered in both academic year-long and summer-long programs at one institution before and during the COVID-19 pandemic (2017–2022). Between two and four students participated in the program at a time. Of the 25 student participants, 22 were at the undergraduate level and 3 were at the postgraduate level. Five students participated from other institutions (three local community colleges and two non-US universities). The Virtual Expedition format facilitated the continued participation of one of the in-person field assistants who collected the majority of the video data for the project. The program leadership and administration was successfully transferred to two PhD students who were familiar with the field but had not previously participated in the specific research project. One student changed their major from Computer Science to Biology after participation in the URE. After the program, >46% were known to continue research in other labs or the lab of the PI and at least six students went on to graduate school in STEM fields.

## Conclusion

Virtual Expeditions are a low-cost, high-benefit research endeavor that addresses fundamental shortcomings of Big Data processing while also broadening participation in a historically exclusionary field. Even without explicit inclusivity design, these experiences attract underrepresented and minoritized students because of their improved accessibility: open source design and virtual research spaces reduce barriers to participation in traditional natural history research. The case studies presented here used video and photographic data of animals, but future research could incorporate other forms of data to engage broader audiences (e.g., overcoming visual impairments by analyzing auditory data, such as frog and bird calls).

Virtual Expeditions are approachable introductory research experiences because even without disciplinary research expertise, students produce research grade data and test real research hypotheses alongside their mentors. Virtual Expeditions make it possible to generate high-quality natural history data at scale using open source technology. Natural history datasets are now large and accessible enough to facilitate the interface of organismal evolutionary and ecological research in novel ways not intended by the original researchers, such as with re-analyses. By leveraging the tools of Big Data, Virtual Expeditions provide one way to facilitate the integration of valuable natural history research into classrooms and interdisciplinary spaces.

## Human subjects research statement

The human subjects research associated with the invertebrate identification CURE was classified as Exempt by both the UC Davis Internal Review Board in 2020 (project ID 1,656,237–1) and the Chapman University Internal Review Board in 2022 (project ID 22–122).

## Supplementary Material

icac065_Supplemental_File

## Data Availability

No data are presented in this commentary article.

## Notes

From the symposium “Open source solutions in experimental design’’ presented at the annual meeting of the Society for Integrative and Comparative Biology virtual annual meeting, January 3–February 28, 2022.
