# Supplementary material for: Virtual Expeditions Facilitated by Open Source Solutions Broaden Student Participation in Natural History Research
Source: Integr Comp Biol. 2022 Jun 6;62(4):1121–30. doi: 10.1093/icb/icac065 (PMC9617212; doi:10.1093/icb/icac065)
Supplement: icac065_Supplemental_File [file icac065_supplemental_file.pdf]

Supplemental Materials:

**Virtual Expeditions broaden student participation in natural history research**

Richelle L. Tanner[1*] & Talia Y. Moore[2*]

1. Chapman University, Environmental Science & Policy Program, Orange, CA 92866

2. University of Michigan, Mechanical Engineering, Robotics Institute, Ecology and Evolutionary Biology, Museum of Zoology. 2505 Hayward St. Ann Arbor, MI 48109

*Co-first authors. Author order was determined by luck in guessing the outcome of a coin flip.

Correspondence: richelle.tanner@richelletanner.com

**Post-training instructions provided as part of the Invertebrate Species ID CURE:**

*Some internal hyperlinks have been removed, but formatting preserved for example purposes.*

You will be inputting your species identifications for each photo at this link. We will be duplicating efforts on photos to start, just to make sure we are all arriving at the same conclusions. This way, you can all gain confidence from each other.

**How do I identify species?**

- Start with the resources provided by William Chan at the USGS. Identifying to group, class, or a higher order classification will help you get started.
- Creating your own dichotomous key from the USGS resources can be very helpful! This is where you use defining characteristics to separate organisms based on single traits. If you haven't heard of a dichotomous key before, see this YouTube video.
- Recent advances in community science platforms online can be very helpful! You may search www.iNaturalist.org based on general location (probably California or West Coast, since it is unlikely to have any reported sightings at our exact field sites)
- There are a number of helpful online resources that are field guides (see example), and it can be helpful to search for databases using key terms such as "Suisun Marsh, benthic, coastal, aquatic, brackish, or macroinvertebrate". However, some of these do not get all the way to species level.
- Here is a field guide to the invertebrates of the San Francisco Bay-Delta
- Here is an interactive map of species found in Suisun Marsh since 1979.
- For your final identification (if you haven't gotten it from a field guide), use the official WoRMS database (world register of marine species): http://www.marinespecies.org

**What if I get stuck?**

- Ask your research partners in your dyad, or in your synchronous zoom room (if we are virtual)
- Ask the research group on Slack in the "id-questions" channel
- If none of us can figure it out, we will solicit external help from other experts

Welcome to the snake behavior project! It's great to have you on board!

Here is your introductory packet of information:

1. You are my connection to the UROP administration, so please keep me informed of any assignments, deadlines, or other items I need to know.
2. Please indicate your availability for our weekly meetings on the attached WhenIsGood poll
3. Please attempt to download and install the software below before our first meeting next week. We can go through it together during the meeting if you get stuck.
4. You should be receiving an invitation to a DropBox folder soon. Please let me know if you do not receive it.

Required software includes:

1. Microsoft Excel
2. Quicktime 7 (not the current version of quicktime) or mpv.io if you have Mac Catalina or newer.
3. Dropbox Desktop Sync (desktop software to automatically download files any time your computer is on)
4. You should be receiving invitations to Slack, Dropbox, and our shared Mendeley bibliography in the next couple of days.

Here is a general procedure for how to score the videos:

Browser actions are in blue, Desktop actions in green

1. In your browser, sync **your temp folder** to your desktop. Don't sync the whole Giant Snake Project, because I can guarantee that you don't have enough space on your computer to do that.

**Location: Giant Snake Project>Individual Information>Paul Temp**

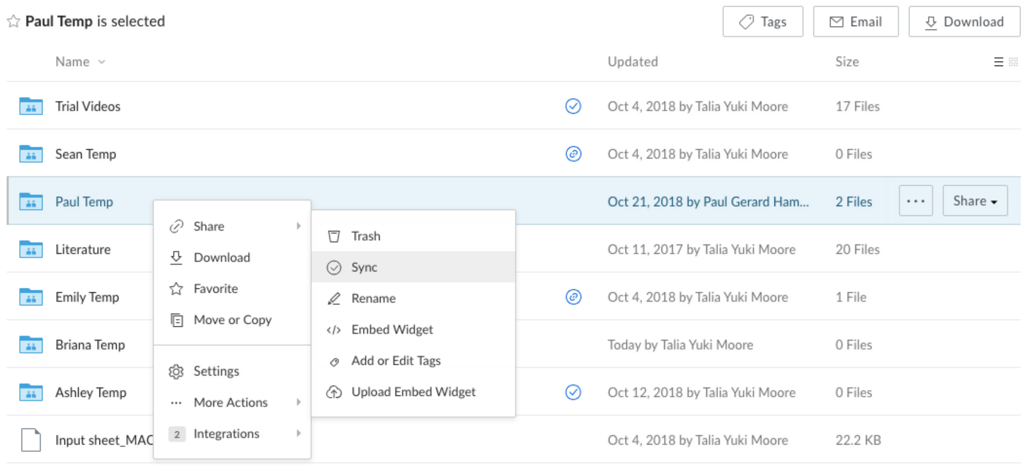

2. In your browser: “Copy” (do not "move") videos from the **Trial Videos** folder into **your temp folder**.

**Location: Giant Snake Project>Individual Information>Trial Videos**

3. On your desktop: Open all videos for a trial and watch them in any video player you like (Quicktime is good). Find out which one is the “best,” meaning which camera can see the snake for most of the time.

4. Open the **template** and click the button to “Enable Macros.” "Save as" the exact same filename as the “best” video ".xlsm" (macro-enabled workbook) into **your temp folder** on your desktop. Copy paste the filename to ensure no misspellings!

5. On your desktop: Use your video player software to watch the video and record the behaviors for each frame according to the diagrams of snake behaviors in the file you just created. You can advance or return to frames using the left and right arrow keys, by sliding the marker along the timeline bar, or by clicking the play/step forward/step backward buttons.

   In the Excel spreadsheet:

   1. HeadFrameIn must be greater than the previous row’s HeadFrameOut.
   2. Use the dropdown menus to select a value for each behavioral column.
   3. When you select a value for the last column, it will copy all behavior values for the next 67 rows.
   4. If you change a value in a row, make sure to select the last column value (even if it remains the same). This re-copies the following rows to the current row
   5. If there are multiple videos for the same trial, they will have the suffix G1, G2, G3 (they’re not synchronized in time).
      a. Pick an event in the trial that can be seen by all cameras. This is your calibration point. For example, if in video G1, the laser shows up at frame 17, and the same thing happens in video G2 in frame 27, then there is a 10 frame difference between the videos.
      b. Pick only one video as your frames of reference, and save the data from all camera angles of one trial to one scoresheet.

6. In your browser: "Move" the data file from **your temp folder** to the **Ethogram data (new system)** folder. This will automatically remove it from your desktop.

**Location: Giant Snake Project>Ethogram data (new system)**

7. On your desktop: Delete the video from **your temp folder** (it should still exist in the **trial videos** folder) to make room for the next videos!

| Date | Data tasks | Meeting Topic | Paper |
|---|---|---|---|
| Week 1 | Install software before meeting | Snake Behavior Instructions | https://doi.org/10.1093/biolinnean/blaa222 |
| Week 2 | Try to score 1 video | Compare data and resolve conflicts | https://doi.org/10.1016/j.cub.2014.11.011 |
| Week 3 | Score half of trial videos | Ask if any cool behaviors observed | https://doi.org/10.1007/BF01968221 |
| Week 4 | Score 2nd half of trial videos | Discuss species/behavior preferences | https://doi.org/10.1371/journal.pone.0080872 |
| Week 5 | Self-assign a dataset | Show the students how to search for papers | https://doi.org/10.1111/j.1558-5646.1993.tb01212.x |
| Week 6 | Self-assigned data collection | Open to new data! | https://doi.org/10.1111/j.1095-8312.1992.tb00642.x |
| Week 7 | Self-assigned data collection | Talk about snake tree and families | https://doi.org/10.1111/j.1439-0310.2004.01034.x |
| Week 8 | Self-assigned data collection | Discuss progress, develop potential hypotheses | https://doi.org/10.1111/j.1558-5646.1992.tb01124.x |
| THANKSGIVING | | | |
| Week 10 | Self-assigned data collection | Discuss progress, develop potential hypotheses | https://doi.org/10.1086/657041 |
| Week 11 | Self-assigned data collection | Discuss progress, develop potential hypotheses | https://doi.org/10.1086/693159 |
| WINTER BREAK+ 1st week of semester | | | |
| Week 12 | Self-assigned data collection | No meeting - scheduling new meeting time | Students lead paper selection and discussion |
| Week 13 | Self-assigned data collection | Set hypotheses in stone, dataset in stone | Students lead paper selection and discussion |
| Week 14 | Self-assigned data collection | Discuss progress, find useful papers to cite | Students lead paper selection and discussion |
| Week 15 | Self-assigned data collection | Discuss progress, find useful papers to cite | Students lead paper selection and discussion |
| Week 16 | Self-assigned data collection | Discuss progress, find useful papers to cite | Students lead paper selection and discussion |
| Week 17 | Self-assigned data collection | Discuss progress, find useful papers to cite | Students lead paper selection and discussion |
| Week 18 | Self-assigned data collection | Start discussing figures, interpretation | Students lead paper selection and discussion |
| SPRING BREAK | | | |
| Week 20 | Statistics and poster design | Discuss the poster template | Focus on student results |
| Week 21 | Statistics and poster design | Finalize Abstracts | Focus on student results |
| Week 22 | Statistics and poster design | Finalize data and statistics | Focus on student results |
| Week 23 | Statistics and poster design | Practice together | Focus on student results |
| Week 24 | | | SYMPOSIUM |
| Week 25 | | | SYMPOSIUM |
